# The impact of herbal medicine in regulating intestinal flora on female reproductive disorders

**DOI:** 10.3389/fphar.2022.1026141

**Published:** 2022-10-14

**Authors:** Min Liu, Jin Yan, Yeke Wu, Hongqiu Zhu, Yefang Huang, Keming Wu

**Affiliations:** ^1^ Department of Gynecology, Hospital of Chengdu University of Traditional Chinese Medicine, Chengdu, Sichuan, China; ^2^ Department of Gynecology, Shaanxi University of Chinese Medicine, Xianyang, China; ^3^ Department of Stomatology, Hospital of Chengdu University of Traditional Chinese Medicine, Chengdu, Sichuan, China; ^4^ School of Medical and Life Sciences, Chengdu University of Traditional Chinese Medicine, Chengdu, Sichuan, China

**Keywords:** herbal medicine, intestinal flora, female reproductive disorders, polycystic ovary syndrome, endometriosis, premature ovarian insufficiency

## Abstract

As an important part of the human intestinal microecology, the intestinal flora is involved in a number of physiological functions of the host. Several studies have shown that imbalance of intestinal flora and its regulation of the intestinal barrier, intestinal immune response, and intestinal flora metabolites (short-chain fatty acids and bile acids) can affect the development and regression of female reproductive disorders. Herbal medicine has unique advantages in the treatment of female reproductive disorders such as polycystic ovary syndrome, endometriosis and premature ovarian insufficiency, although its mechanism of action is still unclear. Therefore, based on the role of intestinal flora in the occurrence and development of female reproduction-related diseases, the progress of research on the diversity, structure and composition of intestinal flora and its metabolites regulated by botanical drugs, Chinese herbal formulas and active ingredients of Chinese herbal medicines is reviewed, with a view to providing reference for the research on the mechanism of action of Chinese herbal medicines in the treatment of female reproductive disorders and further development of new herbal medicines.

## 1 Introduction

The intestinal flora is a symbiotic microorganism colonized in the human intestine, which is an important component of the intestinal microecosystem and is also known as the “second genome” due to its large number, diversity and genetic information, and is involved in regulating metabolic, immune, endocrine and other physiological processes of the host (Blaut and Clavel, 2007; Lynch and Pedersen, 2016). In recent years, with the development of life sciences, the research on human intestinal flora has received extensive attention from scholars worldwide. Under normal circumstances, the intestinal flora is in a dynamic equilibrium under the combined influence of external environmental conditions and host factors, forming a “flora-host” symbiosis. Changes in internal and external factors, such as diet, hormones and inflammation, may contribute to the alterations in the number, type, ratio, location and biological properties of intestinal flora, resulting in imbalance of intestinal flora and disruption of the intestinal barrier, leading to a range of diseases (Schirbel et al., 2013; Zhang et al., 2015). Studies have reported that an inextricable link between intestinal flora imbalance and a variety of diseases, such as obesity (Liu et al., 2017a), type 2 diabetes mellitus (T2DM) (He et al., 2022), metabolic syndrome (Woting and Blaut, 2016), inflammatory bowel disease (Ruigrok et al., 2021), chronic heart disease (Hu et al., 2021) and Alzheimer’s disease (Jiang Y. et al., 2021). Currently, an increasing number of studies have also shown that intestinal flora played a crucial role in the development and regression of female reproductive endocrine disorders such as polycystic ovary syndrome (PCOS) (Rodriguez Paris et al., 2022), premature ovarian insufficiency (POI) (Jiang L. et al., 2021) and endometriosis (EMs) (Svensson et al., 2021). Intestinal flora is involved in various aspects of female reproduction, including follicle and oocyte maturation, fertilization, embryo migration and implantation, and there is a linear correlation between various intestinal flora and serum sex hormone levels (Qi et al., 2021). Therefore, imbalance of intestinal flora may affect female reproductive and endocrine functions, and restoring intestinal flora homeostasis is beneficial to improve female reproductive health and pregnancy outcomes.

The dialectical use of traditional Chinese medicine (TCM) to treat diseases has been practiced in China for thousands of years. Chinese herbal formulas are complex in composition and often consist of combinations of multiple botanical drugs, which are used to reduce toxicity and increase efficacy through complex concoction processes and interactions between botanical drugs (Yin et al., 2022). At the same time, Chinese herbal medicines are rich in natural components with a wide range of action targets, which can provide ideas for the screening and research of new drugs. Chinese herbal medicines are widely used in the treatment of female reproductive disorders (Jiang N. et al., 2020), including PCOS, POI, and EMs, due to their significant efficacy and low side effects. Moreover, herbal medicines can effectively alleviate other concomitant symptoms of female reproductive disorders such as acne, hirsutism, vaginal dryness, and dysmenorrhea (Fang, 2020; Liang et al., 2021; Mao et al., 2022). However, the underlying mechanism of action has not been fully elucidated. Herbal medicines are mostly taken orally into the gastrointestinal tract, and their multiple active ingredients interact directly with intestinal flora to perform the function of regulating the structure of intestinal flora and its metabolites. In addition, intestinal flora can transform the components of herbal medicines to produce secondary metabolites with strong pharmacological activities, such as converting polysaccharide components into short-chain fatty acids (SCFAs) such as propionic acid and butyric acid, which have immunomodulatory effects on human body through fermentation fatty acids to maintain host immune and metabolic homeostasis for disease prevention and treatment (Cockburn and Koropatkin, 2016). Based on this, regulation of intestinal flora dysbiosis may be an important potential target for herbal medicines in the treatment of female reproductive disorders.

Therefore, this paper reviews the mechanism of action of herbal medicines in intervening female reproductive disorders from the perspective of intestinal flora, and introduces natural herbal medicines for treating female reproductive disorders, in order to provide a reference for clinical treatment and further research of herbal medicines for female reproductive disorders.

## 2 Overview of intestinal flora and female reproductive disorders

The intestinal flora is a microbiota parasitic in the human intestine, with a large number of species and a total number of up to 10^14^, which is about 10 times the total number of human cells (de Clercq et al., 2017). The intestinal flora is mainly divided into three major groups: probiotics, pathogenic bacteria and conditionally pathogenic bacteria, mainly composed of the phylum *Firmicutes*, *Bacteroidetes*, *Fusobacteria*, *Proteobacteria* and *Actinobacteria*. Probiotics, together with intestinal mucosal epithelium, intestinal mucus, secretory immunoglobulins, intestine-related lymphoid tissue, bile salts, hormones and gastric acid, form the intestinal mucosal barrier to prevent harmful substances such as bacteria and toxins from entering other tissues, organs and blood circulation in the body through the intestinal mucosa, and regulate body metabolism and immunity. The balance of intestinal flora is a key factor for normal intestinal barrier function. Dysbiosis of intestinal flora impairs the intestinal barrier and decreases the expression of the tight junction proteins occludin and zonula occludin-1 (ZO-1) in the intestinal mucosa, thus promoting increased intestinal permeability and the release of endotoxins into the circulation, which bind to the corresponding receptors to conduct signals, leading to chronic inflammation, insulin resistance (IR) and hyperandrogenemia (HA), affecting immune and metabolic homeostasis (Lam et al., 2012). In addition, SCFAs are metabolites of the intestinal flora and are important components in the regulation of host metabolic activity, with important roles in the regulation of lipid metabolism, feeding control, weight loss and chronic inflammation.

**FIGURE 1:**
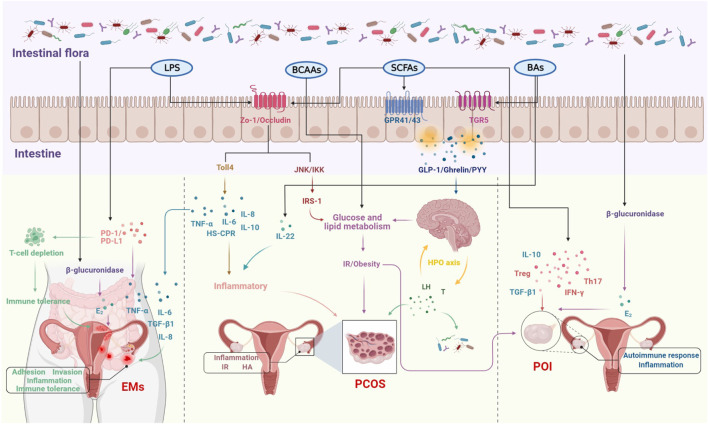
The molecular mechanisms of intestinal flora involved in the development of female reproductive disorders.

In recent years, studies on the relationship between intestinal flora and female reproductive endocrine disorders such as PCOS, POI and EMs have been frequently reported, mainly in terms of correlations, but with the advancement of research techniques, the mechanisms are gradually being investigated. The intestinal flora is involved in a wide range of metabolic activities in the human body and is also known as the “endocrine organ” for maintaining human health, influencing the reproductive endocrine system by regulating hormones, inflammatory factors, metabolism and immunity (Sjogren et al., 2009; Yu and Chen, 2018; Qi et al., 2021). It was reported that estrogen levels decreased significantly following the use of antibiotics, suggesting that intestinal flora may play an important role in estrogen metabolism. The beta (β)-glucuronidase enzyme secreted by the intestinal flora can metabolize estrogen from the bound form to the unbound form. If dysbiosis of the flora results in reduced *ß*-glucuronidase activity, this would lead to a reduction in circulating estrogen, triggering hypoestrogenic changes such as obesity, metabolic syndrome and cognitive decline (Plottel and Blaser, 2011; Franasiak and Scott, 2015). Increasing the number of *ß*-glucuronidase-producing bacteria can lead to an increase in circulating estrogen levels, which can lead to EMs and cancer (Baker et al., 2017). Furthermore, dysbiosis of the intestinal flora leads to disruption of the intestinal barrier, increased intestinal permeability and the entry of lipopolysaccharides into the bloodstream, forming metabolic endotoxemia, which activates inflammatory pathways and releases large amounts of pro-inflammatory factors (Gonzalez et al., 2019), manifesting as a systemic low-grade chronic inflammatory state, and eventually developing a PCOS phenotype such as IR, HA and follicular dysplasia. Similarly, POI has been associated with abnormal lipid and glucose metabolism in addition to inflammation and autoimmune disorders (Ates et al., 2014) and with the development of T2DM (Anagnostis et al., 2019), suggesting that POI may not be a localized lesion of the ovary, but a systemic metabolic disease affecting multiple factors and closely related to the intestinal flora. Therefore, the relative stability of the intestinal flora is important for maintaining female reproductive endocrine health (Figure 1).

## 3 The role of intestinal flora in female reproductive disorders

### 3.1 Intestinal flora in PCOS

PCOS is a complex endocrine metabolic disease characterized by sporadic ovulation or anovulation, HA and polycystic ovary -like changes, affecting 6%–20% of women of reproductive age, clinically manifesting as sporadic menstruation, infertility, hirsutism, acne, IR and obesity, and in the distant future may be complicated by T2DM, hypertension and cardiovascular disease (Escobar-Morreale, 2018). The etiology of PCOS has not been fully elucidated and may be related to genetic, lifestyle and endocrine disorder factors. Several studies have shown that the diversity and relative abundance of intestinal flora in patients with PCOS are different from the general population, inferring that intestinal flora may be related to the etiology of PCOS. The intestinal flora is involved in endotoxemia, SCFAs production, bile acids (BAs) metabolism, amino acid metabolism, and brain-gut peptide secretion, and the above physiopathological processes are associated with the manifestations of HA, IR, chronic inflammatory response, and abnormal brain-gut peptide levels in PCOS (Chen et al., 2020; Jia and Liu, 2021). Therefore, intestinal flora may be involved in the pathogenesis of PCOS through HA, IR, chronic inflammation, and the gut-brain axis, which affect follicular development, sex hormones, and metabolic levels.

#### 3.1.1 Interaction between intestinal flora structure and sex hormone levels in PCOS

There is a two-way interaction between intestinal flora and sex hormones, and intestinal flora may not only be involved in regulating sex hormone levels, but also its diversity and distribution are influenced by sex hormones (Rizzetto et al., 2018; Shin J. H. et al., 2019). Most PCOS studies on the relationship between sex hormones and intestinal flora have focused on serum testosterone (T) levels, with a small number involving estrogen levels. Studies have shown that serum T levels and hirsutism are negatively correlated with the alpha (α) diversity of the intestinal flora. *Bacteroidetes* and *Firmicutes* are two intestinal flora that affect the homeostasis of energy metabolism, and people or animals with obesity or disorders of glucolipid metabolism have more *Firmicutes* and less *Bacteroidetes* (DiBaise et al., 2008). Compared to normal women, PCOS patients have reduced α diversity (Jobira et al., 2020) and increased *ß* diversity (Zeng et al., 2019) in their intestinal flora, with *Bacteroides vulgatus* being strongly associated with PCOS (Qi et al., 2019; Jobira et al., 2020). Compared to control rats, PCOS rats had lower levels of *Lactobacillus*, *Ruminococcus* and *Clostridium*, and higher level of *Prevotella* (Guo Y. et al., 2016). A cross-sectional study involving 46 adolescents found that the diversity and composition of the adolescent intestinal flora were influenced by a combination of gender, sex hormone concentrations, and that sex hormones altered the diversity of the intestinal flora.conversely, intestinal flora is involved in regulating sex hormones (Insenser et al., 2018). A study in which the intestinal flora of adult male mice was transplanted to juvenile female mice resulted in significantly higher T levels in juvenile females, with higher T levels in the germ-lined environment than in the germ-free environment (Markle et al., 2013). This suggested that intestinal flora can affect the secretion of T *in vivo* and can cause endocrine disruption in severe cases. In addition, another study transplanted feces from healthy women and PCOS patients into two groups of mice bodies and showed that mice transplanted with fecal samples from PCOS patients showed PCOS-like manifestations such as IR, increased number of cystic follicles, and elevated levels of T and luteinizing hormone (LH) compared to control mice (Qi et al., 2019). Indicating that dysbiosis of intestinal flora can alter sex hormone levels in the body and trigger PCOS, directly affecting ovarian physiological functions. Recent studies have shown that probiotics can improve intestinal flora disorders and normalize the imbalanced intestinal flora ratio, which in turn improved insulin sensitivity and regulated serum T levels (Zhang F. et al., 2019). Furthermore, estrogen and gut microbiota may act synergistically to influence various aspects of women’s health, including fertility, obesity, diabetes, and cancer.

These results argue for the idea that there is a bidirectional interaction between sex hormone levels and intestinal flora composition and diversity, and provide a basis for further understanding of the relationship between PCOS and intestinal flora.

#### 3.1.2 Disruption of the intestinal barrier triggers endotoxemia promoting chronic inflammation in PCOS

PCOS is a long-term chronic inflammatory disease. It has been suggested that an “intestinal endotoxemia-inflammation mechanism” may be involved in the pathogenesis of PCOS, and that endotoxemia may be involved in the development of PCOS by triggering an inflammatory response (Tremellen and Pearce, 2012). The cell wall fraction lipopolysaccharide (LPS) of Gram-negative bacteria in the intestine, such as *bacterioide* and *Escherichia coli*, is an important factor in the inflammatory activity and early development of metabolic diseases, and has endotoxic effects. Under normal conditions, the intestinal barrier protects the organism from the entry of bacteria, endotoxins and other harmful substances into the blood. However, a long-term high sugar, high fat, low dietary fiber diet can cause dysbiosis and increase the abundance of Gram-negative bacteria, resulting in the destruction of the intestinal mucosal barrier, decreased the expression of ZO-1 and Occludin, increased intestinal permeability, and LPS entering the circulation, resulting in “metabolic endotoxemia”. Compared to normal adult women, PCOS patients have disrupted intestinal flora, impaired intestinal mucosal barrier, increased intestinal permeability, and significantly higher indicators of endotoxemia (Lindheim et al., 2017). Large amounts of LPS into the blood not only activate Toll-like receptor 4 (Toll-4) and recruit the expression of downstream bridging molecules such as tumor necrosis factor α (TNF-α) and inflammatory factors such as interleukin-6 (IL-6), inducing a chronic subclinical inflammatory process. IL-6 and TNF-α are higher in serum and follicular fluid than in healthy women (Amato et al., 2003). In addition, LPS activates the serum kinase c-Jun N-terminal kinase (JNK) and the inhibitor of nuclear factor-κB kinase (IKK) to induce insulin receptor substrate-1 (IRS-1) serine phosphorylation, leading to impairment of the insulin metabolic pathway and triggering IR (Saad et al., 2016; Guadagnini et al., 2019), and hyperinsulinemia may interfere with follicular development, leading to excessive androgen production by follicular theca cells of the ovary, which consequently leads to PCOS. In one study, two groups of mice were fed with normal and high-fat diets for 4 weeks, and compared to control mice, the high-fat group showed signs of obesity and IR, with significantly higher serum LPS concentrations; subsequently, LPS was injected subcutaneously into control mice, which became obese and developed IR after 4 weeks. In addition, our previous study found that elevated fasting insulin and plasma TNF-α, IL-6 and HS-CPR inflammatory factors in PCOS rats were associated with altered intestinal flora (Zhu et al., 2020), which was also confirmed in another study (Lindheim et al., 2017). Supplementation with probiotics can increase the relative abundance of beneficial intestinal bacteria, repair intestinal barrier function, upregulate tight junction protein and mucin synthesis, improve chronic inflammation, and increase insulin sensitivity (Roxas and Viswanathan, 2018; Shin J. et al., 2019). Therefore, when the intestinal mucosal barrier function is impaired, endotoxins produced by the intestinal flora enter the circulation, causing chronic ovarian inflammation and IR and promoting the development of PCOS.

#### 3.1.3 The content of SCFAs affects the occurrence of IR

SCFAs are metabolites of dietary fiber *via* intestinal flora such as *anaerobic bacteria* and *Bifidobacteria*, and are dominated by butyric, propionic and acetic acid, which have important roles in regulating lipid metabolism, IR, chronic inflammation and intestinal immune homeostasis (Guo et al., 2022). SCFAs are important for maintaining intestinal homeostasis and affect intestinal barrier function through various pathways, such as activating the AMPK pathway, increasing expression of tight junction proteins and mucins, to maintain the integrity of the intestinal barrier (Tong et al., 2016). Reduced biosynthesis of SCFAs affects the barrier function of the intestine and increases the translocation of endotoxins in the intestinal wall, thus triggering chronic inflammation and IR, which may lead to the development of PCOS. In addition to stimulating the release of the key hormones Peptide YY (PYY) and Ghrelin in the brain-gut axis, affecting sex hormone-related functions in the hypothalamus and pituitary gland (Zhang J. et al., 2019), SCFAs can also increase insulin levels by binding to G-protein coupled receptor 43 (GPR43) and GPR41 in enteroendocrine cells, intestinal epithelial cells and pancreatic islets, regulating GLP-1 secretion and increasing insulin levels. Dysbiosis of the intestinal flora leads to altered levels of SCFAs, which is more pronounced in PCOS patients with IR. It was shown that the levels of acetate, propionate and butyrate in the intestine of PCOS patients were significantly lower compared to healthy women, and after 10 weeks of probiotic treatment of PCOS patients, the abundance of intestinal *Lactobacillus* was significantly increased, as well as intestinal SCFA levels, which promoted insulin secretion (Zhang J. et al., 2019). Another study showed that increased dietary fiber intake and butyrate supplementation prevented the development of obesity and improved insulin sensitivity (McNabney and Henagan, 2017). The metabolites of intestinal flora, SCFAs, are one of the major signaling pathways between intestinal and host metabolism, and when intestinal flora dysbiosis leads to a decrease in circulating SCFAs, it causes disruption of glucose and lipid metabolism *in vivo*, which may be involved in the pathological process of PCOS.

#### 3.1.4 BAs are involved in improving chronic inflammation and affecting insulin sensitivity

BAs consist of primary bile acids synthesized by the liver and secondary bile acids metabolized by intestinal flora, most of the BAs in the intestinal lumen are absorbed by the distal ileum, and the rest are modified by a variety of intestinal flora and then excreted or passively absorbed. BAs activate glucolipid metabolism and inflammatory signaling pathways, inhibit NLRP3 and immune cell activation by binding to the corresponding receptors thereby ameliorating chronic inflammation, IR and HA (Guo C. et al., 2016). In addition, bile salts can inhibit the proliferation of intestinal pathogenic bacteria, protect intestinal barrier function, and alleviate PCOS-related phenotypes caused by endotoxemia (Das et al., 2019). In a study of the correlation between BAs and HA, glycodeoxycholic acid (GDCA) and taurodeoxy cholic acid (TUDCA) were found to be positively correlated with serum total testosterone (TT) and androstenedione in the PCOS group (Zhang B. et al., 2019). Therefore, altering BAs metabolism may have therapeutic value in PCOS.

Previous studies have shown that a significant increase in the intestinal flora of PCOS patients with *Bacteroides vulgatus* caused to a decrease in the levels of GDCA and TUDCA by inhibiting the reabsorption of BAs, which in turn led to decreased IL-22 secretion. In animal experiments, mice treated with gavage of *Bacteroides vulgatus* or fecal transplants from PCOS patients showed disruption of the motility cycle and polycystic ovary-like changes, accompanied by IR, altered BAs metabolism, reduced IL-22 secretion and infertility. Administration of BAs to different PCOS model mice resulted in significant improvement of ovarian abnormalities and metabolic abnormalities in all mice (Qi et al., 2019). These findings indicate that intestinal flora-mediated BAs metabolism is involved in IL-22 production and regulates inflammatory and glucolipid metabolic processes, which affect ovarian function and insulin sensitivity in PCOS. Thus, dysbiosis of intestinal flora can imbalance BAs synthesis and conversion, affecting lipid digestion and absorption, which in turn affects glucose metabolism and is involved in the development and progression of PCOS.

#### 3.1.5 Branched-chain amino acids affect insulin sensitivity

BCAAs, including leucine, isoleucine and valine, are essential amino acids required by the human body, and their metabolic processes involve various physiological processes such as insulin signaling, fatty acid oxidation, tricarboxylic acid cycle (TCA), glycolysis and mitochondrial oxidation. There is growing evidence that abnormal metabolism of BCAAs is closely associated with IR or metabolic disorders (Karakas et al., 2016; Cunningham et al., 2021), and *Prevotella* is the most effective factor in the intestinal flora for inducing elevated serum BCAAs levels and IR. Serum BCAAs levels were significantly increased in mice on a high-fat diet compared with mice on a normal diet, and a certain degree of IR was also observed (Pedersen et al., 2016). The metabolism of BCAAs in patients with PCOS is similar to that of obese individuals and the level of *Prevotella* in the intestinal flora is elevated in patients with PCOS compared to controls. Therefore, it is likely that the BCAAs pathway of the intestinal flora plays a role in the development of the IR phenotype in PCOS patients (Pedersen et al., 2016). Significant changes in plasma BCAAs were detected in each of the PCOS phenotypes, indicating the presence of abnormal amino acid catabolism and biosynthesis in PCOS patients (Zhao et al., 2012). Furthermore, elevated valine levels and reduced glycine levels were observed in women with PCOS without IR, and these changes were further exacerbated when IR was present in PCOS patients, suggesting that valine and glycine contribute to the development of PCOS not only by influencing IR conditions, but may also be associated with other metabolic disorders, which need to be further explored. In addition, one study added that leucine and valine levels were significantly elevated in follicular fluid of PCOS patients with IR and confirmed that higher levels of BCAA increased the rate of miscarriage and adverse pregnancy outcomes (Zhang et al., 2014). The underlying mechanism may be closely related to BCAAs, which is a metabolic disorder that alters glucose metabolism or induces chronic inflammation that affects insulin sensitivity, exacerbates IR, contributes to the development of PCOS, and affects pregnancy outcome. At present, there are few studies on the relationship between the gut microbiome and BCAA in PCOS patients, and further studies are needed.

#### 3.1.6 Intestinal flora-gut-brain axis disorders affect the development of PCOS

The intestinal flora-gut-brain axis is a bidirectional information network between the intestinal flora and the brain, which consists of the gastrointestinal tract, the enteric nervous system, and the intestinal flora. It is known as the “second brain” of the human body, and plays a role in metabolic diseases such as obesity (Muscogiuri et al., 2019), IR (De Vadder et al., 2014), T2DM (Perry et al., 2016) and PCOS (Zhang J. et al., 2019). The intestinal flora and its metabolites produce including SCFA, brain-gut peptides, neurotransmitters and inflammatory factors (Yano et al., 2015) as initiators of signaling pathways that initially enter the circulation *via* enteroendocrine cells, intestinal chromophores and the immune system or directly through the intestinal mucosal barrier, and subsequently induce central responses by signaling *via* the vagus nerve or the humoral circulation (Perry et al., 2016), thereby controlling the body’s feeding response and the metabolism of lipids, insulin and BAs. Furthermore, sympathetic postganglionic nerves in the gastrointestinal tract regulate the secretion of brain-gut peptides, including Ghrelin, cholecystokinin, glucagon⁃like peptides (GLP) and PYY, which control the absorption of glucose and lipids and the role of human metabolism. SCFA, a metabolite of intestinal flora, is involved in the secretion of GLP-1, Ghrelin, YY peptide and other brain-gut peptides by enteroendocrine cells, activates mammalian rapamycin target protein/signal transduction and transcriptional activator signaling pathways through GPR43 and regulates brain-gut peptides expression (Zhao et al., 2018). Therefore, abnormal metabolism of intestinal flora in turn causes abnormal secretion of peptides, cytokines and inflammatory factors in the intestine. Ghrelin is not only involved in regulating the hypothalamic regulatory nucleus and regulating the secretion of LH; it also affects female reproductive function by delaying the intensity of LH pulse release from the pituitary gland and inhibiting its excessive synthesis and release (Moffett and Naughton, 2020). PYY can also control pituitary gonadotropin secretion *via* neuropeptide two receptors (Pinilla et al., 2007). Therefore, brain intestinal peptides can also affect the hypothalamic ⁃pituitary ⁃ ovarian axis (H-P-O axis) by acting on the central receptors, and then participate in regulating the function of the reproductive system in PCOS. It was found that Ghrelin and PYY were significantly lower in PCOS patients compared with normal women, while serum LH levels and LH/FSH ratio were significantly reduced after successful probiotic transplantation, and improved PCOS-related clinical indicators and increased SCFA levels; while no significant changes were seen in serum LH and SCFA levels in the unsuccessful probiotic transplantation group (Zhang J. et al., 2019). Another PCOS study also showed that Ghrelin and PYY levels were reduced in PCOS patients compared to healthy women and were negatively correlated with waist circumference and androgen levels (Liu et al., 2017b). The mechanism of metformin treatment of PCOS may be related to elevated levels of Ghrelin, tyrosine and GLP-1 (Saydam and Yildiz, 2016). Ghrelin was negatively correlated with *Bacteroides*, and the decrease of Ghrelin caused by PCOS may also be associated with the increase of genus *Bacteroides*. Therefore, as an important bidirectional signaling axis regulating host energy homeostasis and behavior, intervention in the intestinal flora-gut-brain axis may be a new target for the future treatment of PCOS.

In summary, intestinal flora damage the barrier function of the intestinal mucosa through HA, endotoxemia, SCFA, BAs, BCAAs, and the gut-brain axis, activating the body’s chronic inflammatory system and promoting the development of IR, and a vicious circle exists between IR and HA in PCOS patients, further promoting the development of PCOS.

### 3.2 Intestinal flora in EMs

EMs is an estrogen-dependent disorder in which the endometrial glands and mesenchyme grow outside the uterine cavity, affecting 5–10% of women of reproductive age through clinical symptoms such as cyclic bleeding, chronic pelvic pain, difficulty with intercourse and infertility (Giudice and Kao, 2004; Giudice, 2010). The pathogenesis of EMs is complex and has not been fully elucidated. The classical “menstrual reflux theory” suggests that endometrial fragments shed during menstruation leave the uterine cavity *via* the fallopian tube with menstrual blood and grow and develop into ectopic lesions. However, it has been suggested that while menstrual reflux is present in about 90% of healthy women, EMs occur in only 10% (Laschke and Menger, 2016). This suggests that the theory of menstrual reflux cannot fully explain the pathogenesis of endometriosis. Recent studies have revealed that although EMs are benign lesions, they have biological properties similar to malignant tumors, such as infiltration, migration and recurrence. Therefore, some scholars have proposed the “Eutopic endocardium determinism” (Lang, 2020), which suggests that mutations in certain determinants of endometrium cause the development of EMs, complementing the theory of retrograde flow. EMs lesions are mainly located in the pelvic cavity, which is also the site of the intestinal tract. The intestine, which contains a large amount of intestinal flora, plays an important role in the stability of the pelvic environment as an important organ in the pelvic cavity (Rahman-Enyart et al., 2021). It was investigated that EMs rats had altered intestinal microorganisms, as shown by an increase in *Firmicutes/Bacteroidetes* (F/B) ratio and a decrease in the abundance of Ruminococcaceae, and the increase in F/B ratio was strongly associated with inflammation (Cao et al., 2020). Therefore, alterations in the pelvic environment may contribute to the pathogenesis of EMs, and the idea that intestinal flora, as a key regulator of many inflammatory, immune and proliferative diseases, may be involved in the development of EMs has been confirmed in several studies (Ata et al., 2019; Chadchan et al., 2021; Shan et al., 2021; Svensson et al., 2021).

#### 3.2.1 Dysbiosis of intestinal flora elevates circulating estrogen levels

Estrogen plays an important role in maintaining female reproductive development and can induce proliferative diseases such as EMs, uterine fibroids and endometrial cancer by stimulating the proliferation of epithelial cells in the female reproductive tract (Xu et al., 2019; Somasundaram et al., 2020). The intestinal flora is involved in the circulating regulation of estrogen, and dysbiosis increases circulating estrogen levels, stimulating ectopic endometrial invasion and growth with periodic bleeding and pain. The intestinal flora regulates estrogen by secreting *ß*-glucuronidase, which uncouples estrogen into active free estrogen, which is reabsorbed back into the body through the enterohepatic circulation and participates in the regulation of circulating estrogen levels (Dabek et al., 2008; Baker et al., 2017). *ß*-glucuronidase can affect intestinal estrogen metabolism and the growth of hormone-dependent tumors *in vivo* (Ervin et al., 2019). When the intestinal flora is dysbiosis, the increase in *ß*-glucuronidase-producing flora in the intestine causes an increase in circulating estrogen levels, and the balance between circulating estrogen levels and intestinal flora is disrupted, contributing to the invasive growth of ectopic endothelium and leading to the development of EMs or exacerbating their clinical symptoms.

#### 3.2.2 Disturbed intestinal flora promotes massive production of LPS and enhances the adhesion and invasion of ectopic endothelium

It was determined that the abundance of *Bacteroidetes* in the feces of EMs mice was higher than that of normal mice, and after administration of metronidazole treatment, *Bacteroidetes* were not detected in the feces of EMs mice and the ectopic lesions became smaller (Chadchan et al., 2019). Subsequent gavage with fecal bacteria from EMs mice that had previously received metronidazole and had a significant reduction in the size of the ectopic endothelial lesions revealed a significant increase in the size of the endothelial lesions. This suggests that the development of EMs is closely associated with the increase of *Bacteroidetes*. Disturbed intestinal flora can lead to an increase in Gram-negative bacteria, causing a large number of LPS to enter the circulatory system is an important cause of chronic inflammation in humans, and chronic inflammation is prevalent in patients with EMs. A study on rhesus monkeys found that fecal bacteria were significantly altered in the EMs group compared to controls, showing a decrease in *Bifidobacteria* and an increase in Gram-negative bacteria, and that the incidence of intestinal inflammation was higher in the EMs group compared to controls (Bailey and Coe, 2002); this finding is consistent with the results of a randomized controlled trial of women with EMs versus healthy women. EMT also has an important role in the adhesion and invasion of ectopic endothelium in EMs, and is an important factor in the successful implantation of ectopic endothelium as well as in the migration of lesions (Xiong et al., 2015; Xiong et al., 2016). LPS upregulates TLR4 expression, which induces the EMT phenotype and plays an important role in the invasion of ectopic endometrium (Ying et al., 2018). LPS can also induce the expression of adhesion molecules between endometrial and pelvic peritoneal cells, promoting ectopic endometrial adhesion and invasion. According to studies, dysbiosis of intestinal flora in patients with EMs triggers an inflammatory response that leads to an increase in the number of peritoneal macrophages that secrete large amounts of cytokines with fibroblast-promoting properties such as transforming growth factor-beta (TGF-β) (Lindsey and Langhans, 2014). Under inflammatory conditions, TGF-βl not only exhibits pro-adhesive molecular expression effects, promoting adhesion between ectopic endometrial cells and stromal cells; it also has chemotactic effects on macrophages, fibroblasts and neutrophils, promoting the secretion of extracellular matrix such as fibronectin and collagen, leading to the formation of pelvic adhesions (Young et al., 2017). It is hypothesized that intestinal flora plays an important role in the formation of ectopic endothelial adhesions, invasion and pelvic adhesions in patients with EMs by mediating LPS.

#### 3.2.3 Impaired immune clearance of ectopic endothelium due to dysbiosis of the intestinal flora

The observation of continued growth of endometriotic lesions in ovariectomized animals suggests that in addition to ovarian steroid hormones, the innate immune system in the pelvic environment may also regulate the growth of ectopic lesions (Novella-Maestre et al., 2012). The refluxed endometrial tissue as a foreign body in healthy women triggers immune cells in the peritoneal fluid to stimulate an immune response, which results in the clearance of endometrial tissue or cells that reflux with menstrual blood, whereas immune tolerance exists in patients with EMs, and the refluxed endometrial tissue or endometrial cells escape from immune clearance and grow and develop ectopic lesions in the pelvic abdomen (Giudice, 2010). In most EMs studies, immune clearance is very poor despite increased expression of some immune cells in ectopic endothelial tissue, such as CD4^+^ and CD8^+^ (Scheerer et al., 2016), and in-depth studies have revealed overexpression of programmed death receptor-1 (PD-1) and programmed death ligand-1 (PD-L1) on the surface of these immune cells (Wintterle et al., 2003; Zhang H. et al., 2010). On the surface of normal immune cells, PD-1 and PD-L1 show expression levels, but when inflammation is stimulated, the PD-1/PD-L1 signaling pathway is over-activated, inhibiting the activation and proliferation of T cells in the local microenvironment of inflammation, while the killing effect on abnormal cells is greatly reduced and immune tolerance occurs, which in turn decreases the immunity of the body and leads to the appearance of immune escape (Okazaki and Honjo, 2006; Riella et al., 2012; Dai et al., 2014). Thus, the continuous stimulation and activation of the PD-1 pathway by the large number of bacterial endotoxins caused by intestinal flora disorders leads to the overexpression of PD-1 and PD-L1, which induces the depletion of immune T cells and contributes to the development and progression of endometriosis. TNF-α is an immunomodulatory factor secreted by macrophages and T cells with functions of regulating reproductive endocrinology, sperm activity and maintenance of pregnancy, and high levels of TNF-α are detrimental to fertility. Studies have found that dysbiosis of the intestinal flora in patients with EMs combined with infertility subsequently causes a significant increase in the level of TNF-α in the peritoneal fluid, which affects fertilization, implantation and the maintenance of pregnancy (Wang et al., 2018).

Based on the above studies, we learned that intestinal flora may be associated with the development and progression of EMs by affecting the inflammatory, immune or hormonal level and causing alterations in the pelvic environment.

### 3.3 Intestinal flora in POI

POI is defined as the onset of ovarian failure before the age of 40 years, with menstrual arrest (≥4 months), reduced estrogen levels and FSH >25 U/L (2 tests >4 weeks apart) as the main diagnostic criteria, often associated with perimenopausal symptoms, low fertility or even infertility, and an increased risk of osteoporosis and cardiovascular disease affecting 1–2% of women’s reproductive health and quality of life (Fenton, 2015; Luisi et al., 2015; Feng et al., 2022). POI has many causes, including genetic defects, enzyme deficiencies, autoimmune diseases, environmental factors and medical factors such as surgery, chemotherapy or radiotherapy (Sullivan et al., 2016). The microbiological profile of the intestinal microbiome of patients with POI has been found to be altered. Compared to healthy controls, a decrease in the abundance of the Phylum *Firmicutes*, genera *Bulleidia* and *Faecalibacterium* and an increase in the abundance of phylum *Bacteroidetes*, *genera Butyricimonas*, *Dorea*, *Lachnobacterium* and *Sutterella* were observed in the POI group (Wu et al., 2021). Thus, intestinal flora may also be involved in the pathogenesis of POI.

#### 3.3.1 Involvement of intestinal flora in estrogen regulation

The intestinal flora-estrogen axis has a critical impact on the health of menopausal women (Fenton, 2015; Domniz and Meirow, 2019). The intestinal flora affects circulating estrogen levels and the intestinal epithelial estrogen receptor *ß* affects the diversity of the intestinal flora (Ibrahim et al., 2019). Studies demonstrated that menopausal symptoms in ovariectomized rats were associated with reduced estrogen levels after altering the intestinal flora of the rats, while long-term supplementation with a symbiotic preparation containing *Lactobacillus* fermentum and *ß*-glucan prevented menopausal symptoms in rats due to estrogen deficiency (Jeong et al., 2017). Intestinal flora, such as *Firmicutes*, have immunomodulatory effects and influence both estrogen production and metabolism (Fuhrman et al., 2014). Therefore, it is possible that changes in the intestinal flora and its composition and structure are one of the pathogenic mechanisms of POI.

#### 3.3.2 Involvement of intestinal flora in the ovarian immune inflammatory response

The intestinal flora is involved in the development of the intestinal mucosal immune system, promotes the synthesis of intestinal mucosal secretory immunoglobulins, and interacts with intestinal mucosal immune cells to maintain intestinal homeostasis. Autoimmunity accounts for up to 10%–30% of the etiology of POI and is associated with the regulation of various cytokines such as Treg, IFN-γ and Th17 (Jagarlamudi et al., 2010). Intestinal flora can promote Treg cell expression and differentiation, mediate the involvement of Treg cells in anti-inflammatory responses and influence the immune and metabolic homeostasis of the body. SCFAs can regulate Treg development in the human colon. DAPH was found to promote Treg cell development and reduce pro-inflammatory Th17 cell differentiation, reduce colonic inflammation and re-establish immune and metabolic homeostasis in the damaged intestine by increasing the relative abundance of SCFAs-producing bacteria (Ji et al., 2019). POF patients had changes in their Treg numbers and improved immunomodulatory effects after treatment (Song et al., 2018). IFN-γ, a hallmark Th1-type cytokine with autoimmune response-inducing and pro-inflammatory effects, plays an important role in the innate and adaptive immune response of the body. Intestinal flora can influence serum IFN-γ levels and regulate the immune microenvironment, while IFN-γ or genetic stimulation can promote the expression of MHC class II antigens, which may stimulate the autoimmune response and lead to follicular atresia and the development of POF (Coulam and Stern, 1991). A study using hpMSCs transplanted into POF mice found that the decrease in serum TGF-β and the increase in IFN-γ could be reversed, suggesting that the restoration of ovarian function is associated with the production of the relevant cytokines TGF-β and IFN-γ in POF mice Th17 is a subpopulation generated by initial CD4^+^ T cell differentiation that promotes tissue inflammation through the production of IL-17 (Yin et al., 2018b). P.UF1 bacteria from the intestinal flora of breastfed preterm infants can specifically control Th17-Th1 cells *via* IL-10^+^ Treg cells to limit pathogenic bacteria-induced inflammation (Colliou et al., 2018). Accumulating evidence suggests that Th17/Treg homeostasis underlies the pathogenic mechanisms driving autoimmune diseases (Noack and Miossec, 2014; Fasching et al., 2017). The intestinal flora influences the body’s immune function by regulating Th17 cells and thus the ratio of Th17/Tc17 and Th17/Treg cells *in vivo* is altered after POF treatment, and ovarian function is significantly improved (Yin et al., 2018a).

#### 3.3.3 Intestinal flora induced metabolic disturbances leading to POI

Lachnospiraceae, a key flora for intestinal health, is involved in a variety of biological processes such as digestion and metabolism of dietary fibre and carbohydrates and glucose transport (Wu et al., 2019). In a study of tripterygium glycosides -induced DOR rats, reduced intestinal flora diversity and lower abundance of Lachnospiraceae were found (Zhu et al., 2022). Moreover, the abundance of several metabolites, particularly lipids, glycerophospholipids, steroids and amino acids, were significantly altered in the ovarian tissue of POI mice compared to normal mice and were reversed by injection of human umbilical cord mesenchymal stem cells. A meta-analysis showed a correlation between POI and increased risk of T2DM (Anagnostis et al., 2019). Hormone replacement therapy (HRT) significantly reduces atherosclerotic lipid levels in postmenopausal women (Gregersen et al., 2019). The intestinal flora is involved in the development of IR, T2DM by influencing the levels of metabolites (Canfora et al., 2019). The composition of the intestinal flora and the abundance of SCFAs that can be observed in ovariectomized constructs of menopausal rats significantly alter the overall health of the menopausal rats (Zeibich et al., 2021). Multiple lines of evidence suggest that the intestinal flora plays an important role in the development of POI and may influence autoimmune dysfunction (De Luca and Shoenfeld, 2019), bone health (Ibanez et al., 2019), and cognitive and neurological health (Martin et al., 2018). Combined with the ability of the intestinal flora and its metabolites in regulating inflammatory pathway activation, brain-gut peptides secretion and pancreatic *ß* cell destruction (Zheng et al., 2018). Therefore, we hypothesize that changes in the intestinal flora of POI patients lead to metabolic imbalances and subsequently induce menopausal symptoms and other health risks, but further experiments are yet to be conducted to verify this.

Current studies suggest that the relationship between intestinal flora and POI is mainly in terms of autoimmunity and involvement in sex hormone regulation, with some studies suggesting a possible association with metabolic disorders. Intestinal flora can be involved in the regulation of sex hormones either directly or indirectly (Jeong et al., 2017), or by influencing the expression of immune-related cytokines such as Treg, IFN-γ and Th17, or possibly by regulating the body’s metabolic processes to improve POI (Adlercreutz et al., 1984). The relationship between POI and the intestinal flora remains to be investigated.

## 4 Chinese herbal medicine to regulate intestinal flora for female reproductive disorders

Current treatments for female reproductive disorders include lifestyle interventions, pharmacological ovulation treatment and assisted reproductive treatment. Studies have shown that modulation of intestinal flora, glucolipid metabolism, inflammation and immune-related targets can influence the onset and progression of reproductive disorders (Hu et al., 2019; Zhao T. T. et al., 2021; Qi et al., 2021). In particular, therapies that target the regulation of intestinal flora may be one of the effective options for improving reproductive disorders. It has been shown that intestinal flora metabolites, such as SCFAs, can significantly modulate glycolipid metabolism and inhibit the development of inflammation, maintaining female reproductive endocrine homeostasis (Chadchan et al., 2021; Lagowska and Drzymala-Czyz, 2022). Probiotics are intestinal microbial modulators that have been widely used in the treatment of female reproductive endocrine disorders, such as PCOS (Giampaolino et al., 2021), and can effectively improve IR and inflammatory status and reduce ovarian damage in patients with PCOS (Heshmati et al., 2019; Shamasbi et al., 2020).

**FIGURE 2:**
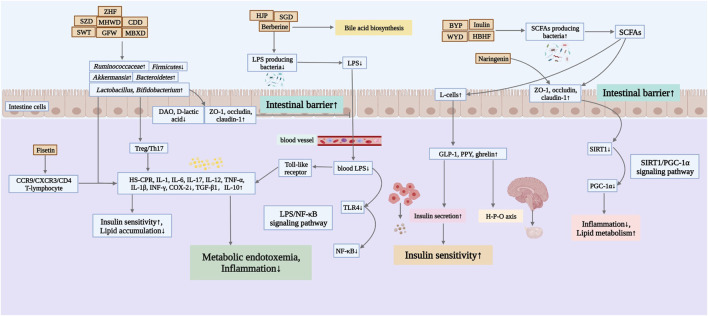
Mechanisms of Chinese herbal medicines in improving female reproductive disorders by regulating the intestinal flora. ZHF, Zhishen Huatan Formula; SZD, Shaofu Zhuyu Decoction; MHWD, Modified Huanglian Wendan Decoction; CDD, Cangfu Daotan Decoction; SWT, Siwu Tang; GFW, Guizhi Fuling Wan; MBXD, Modified Banxia Xiexin Decoction; HJP, Huayu Jiedu Prescription; SGD, Shaoyao-Gancao Decoction; BYP, Buzhong Yiqi Prescription; WYD, Wang’s YiJing Decoction; HBHF, Hashi Bushen Huatan Formula.

Probiotic supplementation in women with PCOS is beneficial in reducing body weight, improving hormone levels and IR, lowering triglyceride (TG) and low density lipoprotein cholesterol (LDL-C) concentrations, increasing sex hormone-binding globulin (SHBG) and improving inflammation and oxidative stress, suggesting that probiotics can modulate inflammation in patients with PCOS (Shoaei et al., 2015; Jamilian et al., 2018). There are some connections between the intestinal flora and the female vaginal flora. Therefore, some scholars have tried to use oral food probiotics to alter the female vaginal flora pattern in a way that inhibits the expression of pro-inflammatory cytokines in the vagina. This action also affects the inflammatory state in the intestine, acting on intestinal flora and immune cells such as macrophages, dendritic cells and intestinal epithelial intrinsic lymphocytes to treat diseases such as EMs and bacterial gynecological inflammation (Ata et al., 2019; Quaranta et al., 2019). Besides, studies have found that probiotics affect FSH levels and reduce body mass in perimenopausal women (Szydlowska et al., 2021). Therefore, targeted regulation of intestinal flora seems to be an effective strategy for the treatment of female reproductive disorders (Qi et al., 2021). Due to its low toxicity and side effects, TCM has become a complementary option for the prevention and treatment of female reproductive disorders (Yang et al., 2022). Over the years, many active ingredients of herbal medicines have been extracted and revealed their important pharmacological effects in anti-inflammatory, antioxidant and reproductive health protection (Yan et al., 2018; Yang et al., 2020; Zhang et al., 2021). Therefore, botanical drugs, Chinese herbal formulas and active ingredients of Chinese herbal medicines have promising applications in the treatment of reproductive disorders associated with disorders of intestinal flora structure (Figure 2).

### 4.1 Botanical drugs

Most Chinese herbal medicines have the function of regulating the disordered intestinal flora while relieving female reproductive endocrine symptoms. It was discovered that *Atractylodes Macrocephala Koidz* [Asteraceae; *Atractylodis macrocephalae rhizoma*] could promote the proliferation of *Bifidobacteria* and *Lactobacillus* in the intestine of mice, reduce the number of harmful bacteria enterobacteria, improve the overall intestinal flora status, and maintain the intestinal microecological balance (Yan et al., 2011). It is speculated that *Atractylodes Macrocephala Koidz.* Might increase the absorption and utilization of glucose by peripheral tissues, especially muscle and adipose tissues, by improving the imbalance of intestinal flora caused by spleen deficiency in patients, protecting the intestinal mucosal barrier, alleviating chronic inflammation, and improving the immunity of the body, and thus improving the IR and inflammatory response.

Puerarin, the main extract of *Pueraria lobata (Willd.) Ohwi* [Leguminosae; *Pueraria Lobata Radix*], could decrease glucose and improve IR by regulating intracellular insulin signaling, increasing cellular Ca-Mg-ATPase activity, and improving the transport of substances such as glucose and insulin in the body (Zhang W. et al., 2010). Puerarin has estrogen-like effects, could regulate glucolipid metabolism, reduce insulin and cholesterol levels, and increase the number of intestinal *Bifidobacteria* and *Lactobacillus,* and decrease the relative abundance of *Escherichia coli* and *Bacteroidetes*, thus exerting a role in reducing androgen synthesis (Xu et al., 2010; Wang, 2016).

The infusion of *Panax ginseng C.A.Mey* (Araliaceae; *Ginsen Radix Et Rhizoma Rubra*) and the extract of *Panax ginseng C.A.Mey.* were able to inhibit the number of ovarian cysts, elevated serum testosterone and E_2_ levels, inhibit the expression levels of macrophage infiltration, pro-inflammatory cytokines IL-1β, IL-6 and inducible nitric oxide synthase (NOS), inhibit NF-κB pathway, and improve PCOS through anti-inflammatory and antioxidant effects (Choi et al., 2020). In addition, treatment of patients with metabolic syndrome with *Panax ginseng C.A.Mey.* revealed significant improvements in serum levels of the biomarkers of lipid metabolism, cholesterol and LDL-C, which might be associated with an increase in *Firmicutes*, *Proteobacteria*, *Bacteroidetes* (Seong et al., 2021).

The main active ingredient of *Salvia miltiorrhiza Bunge* (Lamiaceae; *Salviae miltiorrhizae radix et rhizoma*) is tanshinone, which has estrogen-like biological activity, increases serum LH and FSH levels, and inhibits the overexpression of T and P receptors. It was noted that the diversity index and species abundance of intestinal flora in the salvia miltiorrhiza cell-broken pieces group were significantly higher than those in the control group, and the number of *Bifidobacteria* and *Lactobacillus* also significantly increased (Zeng, 2015). This indicated that Dan Shen wall-breaking tablets had positive regulating power on intestinal microbiota and increased the beneficial bacteria in the intestinal tract. Further studies revealed that the intestinal flora of mice intervened by cryptotanshinone resulted in increased relative abundance of *Ruminococcus* and negatively correlated with inflammatory factors, thus the mechanism of anti-androgenic action of cryptotanshinone and the modulatory effect of intestinal flora were closely related (Wang, 2015; Wang L. et al., 2020). It is hypothesized that *Salvia miltiorrhiza Bunge* can promote the release of intestinal hormones through the regulation of intestinal flora (GLP-1, PYY, etc.), which in turn activates the brain nervous system and induces the pituitary gland to be sensitive to GnRH and increases LH secretion, and inhibits the release of inflammatory factors and improves ovarian function by anti-androgen synthesis but its mechanism of action needs to be verified by more studies.

### 4.2 Chinese herbal formulas

Many modern studies have shown that Chinese herbal formulas can improve the microecological environment of the intestinal tract and thus play a therapeutic role in a variety of female reproductive disorders (Table1).

**TABLE 1:** Changes in the intestinal flora and potential mechanisms.

Intervention	Common composition/Type	Subjects	Changes in intestinal flora	Potential mechanism	Outcome	References
Formulas	Common composition					
Cangfu Daotan Decoction (CDD)	*Atractylodes lan* (Thunb.) DC. [Asteraceae; Atractylodis rhizoma], Cyperus rotund*us L.* [Cyperaceae; *Cyperi rhizoma*], *Citrus × aurantium L.* [Rutaceae; *Aurantii fructus immaturus*], *Arisaema erubescens (Wall.) Schott* [Araceae; *Arisaematis rhizoma*], Citrus × aurantium L. [Rutaceae; Aurantii fructus], *Pinellia ternata (Thunb.) Makino* [Araceae; *Pinelliae rhizoma*], *Conioselinum anthriscoides ‘Chuanxiong’* [Apiaceae; *Chuanxiong rhizoma*], *Poria cocos (Schw.) Wolf* [Polyporaceae; *Poria*], *Shenqu* Purchased by Zhang et al. (2020) from Guangdong Biotechnology Co., Ltd	PCOS-IR patients	*Lactobacillus* and *Bifidobacterium*↑, Enterobacteriaceae↓	-	The cycle ovulation rate and cycle pregnancy rate↑	Zhang and Jiang, (2020)
Cangfu Daotan Decoction (CDD)	*Atractylodes la*ncea (Thunb.) DC. [Asteraceae; *Atractylodis rhizoma*], *Citrus × aurantium L.* [Rutaceae; *Aurantii fructus immaturus*], *Cyperus rotundus L.* [Cyperaceae; *Cyperi rhizoma*], *Arisaema erubescens (Wall.) Schott* [Araceae; *Arisaematis rhizoma*], *Prunus persica (L.) Batsch* [Rosaceae; *Persicae semen*], *Angelica sinensis (Oliv.) Diels* [Apiaceae; *Angelicae sinensis radix*], *Pinellia ternata (Thunb.) Makino* [Araceae; *Pinelliae rhizoma*], *Poria cocos (Schw.) Wolf* [Polyporaceae; *Poria*], *Zingiber officinale Roscoe* [Zingiberaceae; *zingiberis rhizoma recens*], *Citrus × aurantium L.* [Rutaceae; *Aurantii fructus*], *Shenqu*, *Glycyrrhiza glabra L.* [Fabaceae; *Glycyrrhizae radix et rhizoma*] (12:8:10:12:8:15:10:15:5:10:12:8) Prepared by Jiang et al. (2020) according to China Pharmacopoeia	PCOS-IR patients	*Lactobacillus* and *Bifidobacterium*↑, Enterobacteriaceae↓	1. Inflammation: IL-17↓, TGF-β, IL-10↑; 2. Insulin resistance indicators: FBG, 2hPBG, FINS, HOMA-IR↓	-	Jiang et al. (2020a)
Buzhong Yiqi Prescription (BYP)	*Astragalus mongholicus Bunge* [Fabaceae; *Astragali radix*], *Poria cocos (Schw.) Wolf* [Polyporaceae; *Poria*], *Codonopsis pilosula (Franch.) Nannf.* [Campanulaceae; *Codonopsis radix*], *Atractylodes macrocephala Koidz.* [Asteraceae; *Atractylodis macrocephalae rhizoma*], *Actaea cimicifuga L.* [Ranunculaceae; *Cimicifugae rhizoma*], *Bupleurum chinense DC.* [Apiaceae; *Bupleuri radix*], *Citrus × aurantium L.* [Rutaceae; *Aurantii fructus immaturus*], *Angelica sinensis (Oliv.) Diels* [Apiaceae; *Angelicae sinensis radix*], *Acorus tatarinowii Schott* [Araceae; *Acorus tatarinowii rhizoma*], *Salvia miltiorrhiza Bunge* [Lamiaceae; *Salviae miltiorrhizae radix et rhizoma*], *Epimedium sagittatum (Siebold and Zucc.) Maxim.* [Berberidaceae; *Epimedii folium*], *Rehmannia glutinosa (Gaertn.) DC.* [Orobanchaceae; *Rehmanniae radix*] (30:15:15:12:9:6:9:15:15:15:18:18) Prepared by Ni et al. (2021) according to China Pharmacopoeia	PCOS-IR patients	the phylum *Spirochaetae* and the genera *[Eubacterium*]*_rectale_group*, *Escherichia-Shigella*, and *Fusicatenibacter*↑, *Megamonas*↓	1. Insulin resistance indicators: HOMA-IR↓; 2. Fecal metabolites: palmitic, palmitoleic, eicosenoic, erucic, behenic, tetracosanoic, stearic acid and sphingosine 1-phosphate↑, taurocholic acid and xanthine↓	DHEAS, T↓; the cycle ovula	Ni et al. (2021)
Guizhi Fuling Wan (GFW)	*Neolitsea cassia (L.) Kosterm.* [Lauraceae; *Cinnamomi cortex*], *Poria cocos (Schw.) Wolf* [Polyporaceae; *Poria*], *Paeonia × suffruticosa Andrews* [Paeoniaceae; *Moutan cortex*], *Paeonia lactiflora Pall.* [Paeoniaceae; *Paeoniae radix rubra*], Prunus persica (L.) Batsch [Rosaceae; Persicae semen] Purchased by Zhu et al. (2020) from Chengdu Jiuzhitang Jinding Pharmaceutical Co., Ltd	PCOS SD rats	*Alloprevotella*, *Firmicutes*, Ruminococcaceae *UCG-003*, and Lachnospiraceae *UCG-008*↑, *Bacteroidetes*, *Proteobacteria*↓	1. Inflammation: TNF-α↓; 2. Insulin resistance indicators: FPG, FINS and HOMA-IR↓	-	Zhu et al. (2020)
Modified Banxia Xiexin Decoction (MBXD)	*Pinellia ternata (Thunb.) Makino* [Araceae; *Pinelliae rhizoma*], *Scutellaria baicalensis Georgi* [Lamiaceae; *Scutellariae radix*], *Coptis chinensis Franch.* [Ranunculaceae; *Coptidis Rhizoma*], *Zingiber officinale Roscoe* [Zingiberaceae; *Zingiberis rhizoma recens*], *Codonopsis pilosula (Franch.) Nannf.* [Campanulaceae; *Codonopsis radix*], *Glycyrrhiza glabra L.* [Fabaceae; *Glycyrrhizae radix et rhizoma*], *Ziziphus jujuba Mill.* [Rhamnaceae; *Jujubae fructus*], *Epimedium sagittatum (Siebold and Zucc.) Maxim.* [Berberidaceae; *Epimedii folium*], *Lycium barbarum L.* [Solanaceae; *Lycii fructus*] (9:20:10:9:12:12:9:15:30) Prepared by Zhao et al. (2022) according to China Pharmacopoeia	PCOS SD rats	phyla *Verrucomicrobiota Proteobacteria* and genera *Akkermansia* and *Blautia*↑, genus *Clostridium_sensu_stricto_1*, *Firmicutes* and *Actinobacteriota*↓	1. Inflammation: HS-CPR, IL-6 and TNF-α↓; 2. Insulin resistance indicators: HOMA-IR↓	body weight↓	Zhao et al. (2022)
Hashi Bushen Huatan Formula (HBHF)	*Cuscuta chinensis Lam.* [Convolvulaceae; *Cuscutae semen*], *Ziheche*, *Poria cocos (Schw.) Wolf* [Polyporaceae; *Poria*], *Pueraria lobata (Willd.) Ohwi* [Leguminosae; *Pueraria Lobata Radix*], *Shinanye*, *Salvia miltiorrhiza Bunge* [Lamiaceae; *Salviae miltiorrhizae radix et rhizoma*], *Atractylodes lancea (Thunb.) DC.* [Asteraceae; *Atractylodis rhizoma*], *Gynochthodes officinalis (F.C.How) Razafim. and B.Bremer* [Rubiaceae; *Morindae officinalis radix*], *Rehmannia glutinosa (Gaertn.) DC.* [Orobanchaceae; *Rehmanniae radix*], *Epimedium sagittatum (Siebold and Zucc.) Maxim.* [Berberidaceae; *Epimedii folium*], *Gleditsia sinensis Lam.* [Fabaceae; *Gleditsiae spina*], *Lujiao*, *Gentiana scabra Bunge* [Gentianaceae; *Gentianae radix et rhizoma*], *Senna tora (L.) Roxb.* [Fabaceae; *Cassiae semen*], *Crataegus pinnatifida Bunge* [Rosaceae; *Crataegi fructus*], *Alisma plantago-aquatica subsp. orientale (Sam.) Sam.* [Alismataceae; *Alismatis rhizoma*], *Nelumbo nucifera Gaertn.* [Nelumbonaceae; *Nelumbinis folium*], *Plantago asiatica L.* [Plantaginaceae; *Plantaginis semen*] (20:6:15:15:15:30:10:15:15:15:15:15:10:10:15:15:10:15) Prepared by Wu et al. (2022) according to China Pharmacopoeia	PCOS SD rats	-	1. Inflammation: IL-1β↓; 2. Insulin resistance indicators: FINS, HOMA-IR↓, Adiponectin receptors in ovarian and adipose tissues ↑; 3. SCFAs: butyric acid↓, acetic acid and propionic acid↑	-	Wu et al. (2022b)
Wang’s YiJing Decoction (WYD)	*Dipsacus asper Wall. ex DC.* [Caprifoliaceae; *Dipsaci radix*], *Eucommia ulmoides Oliv.* [Eucommiaceae; *Eucommiae cortex*], *Viscum coloratum (Kom.) Nakai* [Santalaceae; *visci herba*], *Gynochthodes officinalis (F.C.How) Razafim. and B.Bremer* [Rubiaceae; *Morindae officinalis radix*], *Cuscuta chinensis Lam.* [Convolvulaceae; *Cuscutae semen*], *Pinellia ternata (Thunb.) Makino* [Araceae; *Pinelliae rhizoma*], *Citrus × aurantium L.* [Rutaceae; *Aurantii fructus immaturus*], *Poria cocos (Schw.) Wolf* [Polyporaceae; *Poria*], *Atractylodes macrocephala Koidz.* [Asteraceae; *Atractylodis macrocephalae rhizoma*], *Citrus × aurantium L.* [Rutaceae; *Aurantii fructus*], *Artemisia capillaris Thunb.* [Asteraceae; *Artemisiae scopariae herba*], *Bupleurum chinense DC.* [Apiaceae; *Bupleuri radix*], *Angelica sinensis (Oliv.) Diels* [Apiaceae; *Angelicae sinensis radix*], *Cyathula officinalis K.C.Kuan* [Amaranthaceae; *Cyathulae radix*] (15:15:15:15:15:10:10:15:30:10:10:6:10:10) Prepared by Chang et al. (2022) according to China Pharmacopoeia	PCOS-IR patients	-	1. Insulin resistance indicators: HOMA-IR↓; 2. Gut-brain axis: ghrelin, PYY, GLP-1↑; 3. SCFAs: SCFAs in fresh stool↑	T, DHT, FAI, AMH, E2, LH, FSH and the bilateral ovarian volume↓, SHBG↑	Chang et al. (2022)
Modified Huanglian Wendan Decoction (MHWD)	*Coptis chinensis Franch.* [Ranunculaceae; *Coptidis Rhizoma*], *Scutellaria baicalensis Georgi* [Lamiaceae; *Scutellariae radix*], *Bambusa tuldoides Munro* [Poaceae; *Bambusae caulis in taenias*], *Citrus × aurantium L.* [Rutaceae; *Aurantii fructus immaturus*], *Poria cocos (Schw.) Wolf* [Polyporaceae; *Poria*], *Citrus × aurantium L.* [Rutaceae; *Aurantii fructus immaturus*], *Cyperus rotundus L.* [Cyperaceae; *Cyperi rhizoma*], *Pinellia ternata (Thunb.) Makino* [Araceae; *Pinelliae rhizoma*], *Salvia miltiorrhiza Bunge* [Lamiaceae; *Salviae miltiorrhizae radix et rhizoma*], *Angelica sinensis (Oliv.) Diels* [Apiaceae; *Angelicae sinensis radix*], *Cuscuta chinensis Lam.* [Convolvulaceae; *Cuscutae semen*], *Atractylodes macrocephala Koidz.* [Asteraceae; *Atractylodis macrocephalae rhizoma*] (6:10:15:12:15:12:12:10:12:12:24:15) Prepared by Zhou et al. (2021) according to China Pharmaco poeia	PCOS-IR patients	*Prevotella* and *Bifidobacterium*↑, *Bacteroides* and *Escherichia coli*↓	1. Inflammation: IL-1, IL-6, TNF-α↓; 2. Insulin resistance indicators: FBG, FINS, HOMA-IR↓	BMI, WHR↓; the recovery rate of menstrual and ovulation↑	Zhou et al. (2021)
Shaoyao-Gancao Decoction (SGD)	*Paeonia lactiflora Pall.* [Paeoniaceae; *Paeoniae radix alba*], *Glycyrrhiza glabra L.* [Fabaceae; *Glycyrrhizae radix et rhizoma*] (1:1) Prepared by Chang et al. (2021) according to China Pharmacopoeia	PCOS SD rats	*Firmicutes/Bacteroidetes* ratio and *Turicibacter*↓, *Akkermansia*, *Blautia*, *Bacteroides*, *Coprococcus* and *Butyricicoccus*↑	1. Inflammation: LPS, IL-18, IL-1β, IL-6 and TNF-α↓; 2. Insulin resistance indicators: HOMA-IR↓; 3. Intestinal barrier: occluding and claudin-1↓; 4. Signaling pathway: TLR4/NF-κB↓	T, LH↓, E_2_, FSH↑; cystic follicles↓, corpus luteum↑	Chang et al. (2021)
Shaoyao-Gancao Decoction (SGD)	*Paeonia lactiflora Pall.* [Paeoniaceae; *Paeoniae radix alba*], *Glycyrrhiza glabra L.* [Fabaceae; *Glycyrrhizae radix et rhizoma*] (1:1) Prepared by Deng et al. (2021) according to China Pharmacopoeia	PCOS SD rats	*Firmicutes, Firmicutes/Bacteroidetes* ratio, *Lactobacillus* and *Odoribacter*↓, *Bacteroidetes*↑	1. Plasma metabolites: Leucine, glutamine, tryptophan, proline, uric acid, and adenosine↑, lauric, palmitic, linoleic acid and acetylcarnitine↓; 2. Signaling pathway: Linoleic acid metabolism, alanine, aspartate and glutamate metabolic pathways	T↓; Body weight and ovarian weight↓	Deng et al. (2021)
Zhishen Huatan Formula (ZHF)	*Bupleurum chinense DC.* [Apiaceae; *Bupleuri radix*], *Paeonia lactiflora Pall.* [Paeoniaceae; *Paeoniae radix alba*], *Angelica sinensis (Oliv.) Diels* [Apiaceae; *Angelicae sinensis radix*], *Cyperus rotundus L.* [Cyperaceae; *Cyperi rhizoma*], *Citrus × aurantium L.* [Rutaceae; *Aurantii fructus*], *Conioselinum anthriscoides ‘Chuanxiong’* [Apiaceae; *Chuanxiong rhizoma*], *Glycyrrhiza glabra L.* [Fabaceae; *Glycyrrhizae radix et rhizoma*], *Atractylodes la*ncea (Thunb.) DC. [Asteraceae; Atractylodis rhizoma], Citrus × *aurantium L.* [Rutaceae; *Aurantii fructus immaturus*], *Poria cocos (Schw.) Wolf* [Polyporaceae; *Poria*], *Pinellia ternata (Thunb.) Makino* [Araceae; *Pinelliae rhizoma*], *Coix lacryma-jobi var. Ma-yuen (Rom.Caill.) Stapf* [Poaceae; *Coicis semen*], *Scutellaria baicalensis Georgi* [Lamiaceae; *Scutellariae radix*], *Cullen corylifolium (L.) Medik.* [Fabaceae; *Psoraleae fructus*], *Zishiying* (10:15:15:12:15:10:6:15:10:15:12:15:10:10:10) Prepared by Li et al. (2021) according to China Pharmacopoeia	PCOS-IR patients	*Lactobacillus* and *Bifidobacteria*↑, Enterobacteriaceae and *Enterococci*↓	1. Inflammation: CRP, IL-6 and TNF-α↓; 2. Insulin resistance indicators: FINS, FPG, HOMA-IR↓; 3. Intestinal barrier: DAO, D-lactic acid↓; 4. Lipid metabolism indicators: TC, TG↓, HDL-C↑	-	Li et al. (2021)
Siwu Tang (SWT)	*Angelica sinensis (Oliv.) Diels* [Apiaceae; *Angelicae sinensis radix*], *Conioselinum anthriscoides ‘Chuanxiong*’ [Apiaceae; *Chuanxiong rhizoma*], *Paeonia lactiflora Pall.* [Paeoniaceae; *Paeoniae radix alba*], *Rehmannia glutinosa (Gaertn.) DC.* [Orobanchaceae; *Pehmanniae radix praeparata*] (1:1:1:1) Prepared by Zhu et al. (2022) according to China Pharmacopoeia	DOR SD rats	*Firmicutes* and *Bacteroidetes*↓	1. Signaling pathway: Carbohydrate transport and metabolism, Amino acid transport and metabolism	FSH, LH↓, E2, P↑	Zhu et al. (2022)
Huayu Jiedu Prescription (HJP)	*Sargentodoxa cuneata (Oliv.) Rehder and E.H.Wilson* [Lardizabalaceae; *Sargentodoxae caulis*], *Curcuma aromatica Salisb.* [Zingiberaceae; *Curcumae rhizoma*], *Typha angustifolia L.* [Typhaceae; *Typhae pollen*], *Boswellia sacra Flück.* [Burseraceae; *Olibanum*], *Commiphora myrrha (T.Nees) Engl.* [Burseraceae; *Myrrha, gummi-resina*], *Epimedium sagittatum (Siebold and Zucc.) Maxim.* [Berberidaceae; *Epimedii folium*] (30:9:9:9:9:18)	EMs C57BL/6J mice	*Proteobacteria*, *Verrucomicrobia*, *Parasutterella*, *Akkermansia* and *Allobaculum*↓, *Cyanobacteria*, *Lactobacillus*, Lachnospiraceae*_NK4A136_group*↑	1. Inflammation: LPS↓; 2. Fecal metabolites: homoveratric acid, melilotoside C, physapubescin↑; 3. Signaling pathway: linoleic acid metabolism, Toll-like receptor	Vimentin, E-cadherin↓	Zhao et al. (2021)
Shaofu Zhuyu Decoction (SZD)	*Foeniculum vulgare Mill.* [Apiaceae; *Foeniculi amari fructus*], *Zingiber officinale Roscoe* [Zingiberaceae; *Zingiberis rhizoma recens*], *Corydalis yanhusuo (Y.H.Chou and Chun C.Hsu) W.T.Wang ex Z.Y.Su and C.Y.Wu* [Papaveraceae; *Corydalis rhizoma*], *Commiphora myrrha (T.Nees) Engl.* [Burseraceae; *Myrrha, gummi-resina*], *Angelica sinensis (Oliv.) Diels* [Apiaceae; *Angelicae sinensis radix*], *Conioselinum anthriscoides ‘Chuanxiong’* [Apiaceae; *Chuanxiong rhizoma*], *Neolitsea cassia (L.) Kosterm.* [Lauraceae; *Cinnamomi cortex*], *Paeonia lactiflora Pall.* [Paeoniaceae; *Paeoniae radix rubra*], *Typha angustifolia L.* [Typhaceae; *Typhae pollen*], *Wulingzhi* Prepared by Cao et al. (2020) according to China Pharmacopoeia	EMs SD rats	*Firmicutes*/*Bacteroidetes* ratio↓, Ruminococcaceae↑	1. Inflammation: COX-2↓; 2. Gut barrier	-	Cao et al. (2020)
Active ingredients	Type					
Berberine	Alkaloid	PCOS SD rats	*Bacteroidetes*, *Proteobacteria*, *Actinobacteria*, *Verrucomicrobia*, *Bacteroides*, *Lactobacillus*, *Bifidobacterium* and *Sutterella*↑, *Firmicutes*, *Prevotella*, *Oscillospira*, *Oscillospira* and *Anaeroplasma*↓; Gram-Negative and Potentially Pathogenic↓	1. Inflammation: LPS, IL-1β, INF-γ, TNF-α↓; 2. Insulin resistance indicators: HOMA-IR↓; 3. Signaling pathway: LPS/NF-κB↓	LH, T, LH/FSH ratio and body weight↓	Zhao et al. (2022)
Berberine	Alkaloid	PCOS SD rats	*Firmicutes*↓, *Bacteroidetes*↑	1. Insulin resistance indicators: HOMA-IR↓; 2. Metabolites: glutamine↑, unsaturated acids [CH = CH], and glucose↓	T↓	Shen et al. (2021)
Inulin	polysaccharide	PCOS C57BL/6J mice	*Bifidobacterium*↑, *Parasutterella*, *Helicobacter* and *Proteobacteria*↓	1. Inflammation: TNF-α, IL-6 and IL-17A↓	T and body weight↓, E_2_↑	Xue et al. (2019)
Inulin	polysaccharide	PCOS C57BL/6J mice	*Lactobacillus*, *Bifidobacterium*, Prevotellaceae and *Akkermansia*↑, *Anaerotruncus*↓	1. Inflammation: IL-6, TNF-α↓, IL-22↑; 2. Signaling pathway: bile acid biosynthesis	LH, T↓, FSH, E_2_, P, PRL↑	Li et al. (2022)
Naringenin	flavonoid	PCOS SD rats	*Ge*mella, Prevotell, Veillon*ella* and *Fusobacterium*↓, the beneficial bacterial genera of *Blautia, Helicobacter*, *Ruminococcus, Lactobacillus*, *Coprococcus, Parabacteroides*, *Faecalibacterium, Roseburia*, *Butyricicoccus, Streptococcus* and *Paraprevotella*↑	1. Insulin resistance indicators: FBG, FINS, HOMA-IR and ISI↓; 2. Gut barrier: occludin, claudin-1↑; 3. Signaling pathway: SIRT1/PGC-1α↓, Carbohydrate metabolism, Amino acid metabolism, Lipid metabolism	LH, T, LH/FSH ratio and weight↓	Wu et al. (2022a)
Fisetin	bioflavonoid	POI C57BL/6J mice	*uncultured_bacterium_f_*Lachnospiraceae↑, *Akkermansia*↓	1. Inflammation: CCR9/CXCR3/CD4 T-lymphocyte, IL-12↓; 2. Signaling pathway: carbohydrate and nucleotide metabolism↑, lipid and amino acid metabolism↓	AMH↑	Li et al. (2021)

Disturbances in intestinal flora and fecal metabolites are closely associated with HA and IR, and treatment with a combination of Chinese herbal medicines that tonify kidney and strengthen spleen can achieve better benefits. Buzhong Yiqi prescription (BYP) improved HA in PCOS patients by modulating the abundance of *Bacteroides*, (*Eubacterium*)*_ventriosum_group* and its differential fecal metabolites. (Ni et al., 2021). Wang’s YiJing decoction (WYD), with tonifying kidney and strengthening spleen as the main treatment, could significantly improve endocrine metabolic disorders and sex hormone levels in PCOS-IR patients, possibly related to the regulation of the gut-brain axis (Chang et al., 2022). The improvement of PCOS-IR by modified Banxia Xiexin decoction (MBXD) could be achieved by regulating the disorder of intestinal microbiota and thus improving the metabolic disorder of the body, while *Akkermansia* might play an important role in the treatment (Zhao et al., 2022).

Studies have shown that formulas are essential for reshaping the structure of intestinal flora and maintaining intestinal immune balance. After the intervention with Cangfu Daotan decoction (CDD), the proportion of *Bifidobacterium* and *Lactobacillus* increased and the relative abundance of Enterobacteriaceae decreased in PCOS patients, while the ovulation and cycle pregnancy rate increased and hormonal abnormalities significantly improved, suggesting that the control of PCOS phenotypes by CDD may be achieved by regulating the structure of the intestinal flora, promoting the growth of dominant bacteria such as *Bifidobacterium* and *Lactobacillus* in the intestinal flora, and inhibiting conditionally pathogenic bacteria such as Enterobacteriaceae (Zhang and Jiang, 2020). This conclusion was verified in another study, which also showed that the combination of CDD with other therapies in obese PCOS patients could reduce IR, correct Treg/Th17 imbalance, modulate T-cell immune response, and improve imbalance of gut microbial homeostasis with fewer adverse effects (Jiang X. L. et al., 2020). In addition, the Chinese herbal formula modified Huanglian Wendan decoction (MHWD), which combined resolving phlegm and tonifying kidney, effectively regulated intestinal flora, increased *Prevotella* and *Bifidobacterium*, decreased *Bacteroides* and *Escherichia coli*, reduced IL-1, IL-6 and TNF-α inflammatory factor levels, improved glucose metabolism and IR, and promoted menstruation and ovulation recovery (Zhou et al., 2021). Another study also showed that *Lactobacillus* and *Bifidobacteria* were negatively correlated with intestinal barrier function, glucose metabolism, inflammatory factors and TC and TG, and positively correlated with HDL-C. Zhishen Huatan Formula (ZHF) effectively increased the abundance of *Lactobacillus* and *Bifidobacteria*, improved intestinal barrier function, alleviated inflammatory response, and corrected disorders of glucose and lipid metabolism, and had therapeutic effects on obese PCOS patients (Li et al., 2021). Hashi Bushen Huatan Formula (HBHF) may improve PCOS-IR by promoting intestinal SCFAs production, decreasing pro-inflammatory factor secretion, and increasing lipocalin receptor expression (Wu L. L. et al., 2022).

A classical formula based on harmonizing the liver and spleen, Shaoyao-Gancao decoction (SGD) reconstructed the structure of intestinal flora in PCOS rats, increased the abundance of *Akkermansia Blautia*, *Bacteroides*, *Butyricoccus* and *Coprococcus*, decreased the F/B ratio and LPS-producing pathogens *Proteobateria* abundance, reduced pro-inflammatory factors, increased the expression of ocludin and claudin1, protected the intestinal barrier to reduce LPS transport, and inhibited TLR4/NF-κB signaling pathway to improve the inflammatory response in PCOS rats (Chang et al., 2021), and the mechanism of action might be related to the regulation of endogenous metabolites and the improvement of intestinal flora disorders in PCOS (Deng et al., 2021). The decrease in intestinal function caused by altered intestinal flora is closely related to the drug process *in vivo*. It was found that intestinal flora affected the metabolic and kinetic changes of SGD in PCOS rats, playing a role in reducing adverse effects and enhancing pharmacological activity (Cheng, 2019). Treatment with the herbal formula Siwu Tang (SWT), which nourishes Blood and invigorates Blood, resulted in a downregulation of FSH levels, an increase in E_2_, and an increase in intestinal flora diversity in DOR rats, with *Firmicutes* and *Bacteroidetes* being the dominant flora (Zhu et al., 2022), and it is hypothesized that the improvement of reproductive function with the herbal formula may be related to the regulation of the abundance of *Firmicutes* and *Bacteroidetes*.

The use of herbal formulas based on activating blood circulation and resolving blood stasis in reproductive disorders is also widespread, and their mechanism of action may also involve changes in intestinal flora. A previous study found that Guizhi Fuling Wan (GFW) induced an increase in the alpha diversity of the intestinal flora of PCOS-IR rats, as evidenced by an increase in the relative abundance of Lachnospiraceae *UCG-008*, Ruminococcaceae *UCG-003* and *Alloprevotella*, to improve inflammation and correct IR (Zhu et al., 2020). In another study, after 4 weeks of Shaofu Zhuyu decoction (SZD) treatment of EMs rats, a normalization of the intestinal microbiota, a decrease in the ratio of F/B and an increase in the abundance of Ruminococcaceae were observed, restoring the damaged intestinal barrier function and alleviating the inflammatory response in the ectopic endometrial tissue and pelvis (Cao et al., 2020). In addition, Huayu Jiedu Prescription (HJP) significantly reduced the level of inflammatory factors in the peritoneal fluid of EMs, remodeled or even reversed some of the altered intestinal flora and intestinal metabolites, reduced the level of LPS *in vivo* and attenuated ectopic focal fibrosis to improve the reproductive disorders caused by EMs (Zhao Q. Q. et al., 2021).

### 4.3 Active ingredients of Chinese herbal medicines

Experimental studies have shown that many active ingredients in Chinese herbal medicines including alkaloids, flavonoids, triterpenoids, polyphenols and polysaccharides have the ability to improve the disorders of intestinal flora during reproductive disorders (Table 1).

Berberine is a quaternary alkaloid isolated from *Coptis chinensis Franch* [Ranunculaceae; *Coptidis Rhizoma*]. Modern research has shown that berberine improves menstrual cycles, ovulation rates, endocrine abnormalities and metabolic dysregulation in PCOS. Berberine indirectly improved IR, regulated hormone secretion and increased ovulation and conception rates in PCOS patients mainly by reducing the relative abundance of polysaccharide flora, increasing the relative abundance of SCFAs producing flora and reducing endotoxemia (Wang T. et al., 2017). Additionally, berberine inhibited the relapse of EMs by affecting the concentration of metabolites involved in energy homeostasis such as glucose, glutamine and lactate (Warowicka et al., 2021). Several studies have suggested that the mechanism of action of berberine in reducing HA and hyperinsulinemia in PCOS model rats may be related to the promotion of SCFAs production by inhibiting the LPS/NF-κB signaling pathway, increasing the relative abundance of beneficial intestinal bacteria and decreasing the relative abundance of pathogenic bacteria (Zhao et al.; Shen et al., 2021). Thus, berberine modulation of intestinal flora may improve the metabolic and inflammatory state of the organism and be beneficial for reproductive function.

Glycyrrhizin and total saponins of glycyrrhizin are triterpenoids extracted from *Glycyrrhiza glabra L* [Fabaceae; *Glycyrrhizae radix et rhizoma*] with anti-inflammatory effects that could improve ovarian morphology, reduce androgen secretion levels and enhance insulin sensitivity in a letrozole-induced PCOS rat models (Jiang L. et al., 2020). In addition, glycyrrhizin significantly inhibited LPS-induced levels of inflammatory mediators such as TNF-α, IL-1β, NO and PGE_2_ and attenuated the endometrial inflammatory response, making it a potential herb for the treatment of EMs (Wang X. R. et al., 2017). The total saponins of glycyrrhizin was able to significantly restore intestinal flora diversity, promote the growth of beneficial bacteria and maintain intestinal environmental homeostasis, suggesting that its ability to reduce serum testosterone, inflammation levels and improve ovarian function was associated with intestinal flora (Wang J. et al., 2020).

Studies have found that inulin, a polysaccharide extracted from plants, could increase *Bifidobacterium* and reduce *Parasutterella*, *Helicobacter* and *Proteobacteria*, alleviating PCOS by regulating intestinal microbiology and alleviating inflammation, suggesting that inulin has potential clinical application for the treatment of PCOS (Xue et al., 2019). Similar results were obtained in a study of supplementation with yogurt enriched with Inulin for the treatment of PCOS, and it was also suggested that yogurt enriched with Inulin alleviated reproductive dysfunction in PCOS mice by modulating the gut microbiota and BAs profile (Li et al., 2022).

Fisetin, a natural bioflavonoid found in abundance in many fruits and vegetables, has antioxidant and anticancer properties and has recently been found to significantly reverse ovarian damage in POF mice. It was revealed that fisetin supplementation significantly increased the abundance of genera *uncultured_bacterium_f_*Lachnospiraceae and significantly decreased the abundance of *Akkermansia* in POI mice, and significantly reduced the number of CCR9/CXCR3/CD4 T lymphocytes and CD4/IL-12 cells in peripheral blood significantly decreased (Lin et al., 2020). It was shown that fisetin has a therapeutic effect on POF by modulating the structure and distribution of intestinal flora in POF mice and reducing peripheral blood CCR9/CXCR3/CD4 T lymphocyte counts and IL-12 secretion to alleviate the ovarian inflammatory microenvironment. Naringenin, a citrus flavonoid, was recently found to have beneficial effects in the treatment of PCOS by upregulating the expression of PGC-1α, SIRT1, occludin and claudin-1 in the colon and downregulating the abundance of *Prevotella* and *Gemera* to improve the intestinal mucosal barrier and glucose metabolic pathways in PCOS rats (Wu Y. X. et al., 2022). Furthermore, quercetin is also a class of flavonoid natural compounds with anti-inflammatory, antioxidant, anti-obesity and anti-cancer properties. Studies have shown that quercetin can intervene in the development of PCOS, EMs and POI in a multi-targeted and multi-pathway manner (Park et al., 2019; Chen et al., 2022; Zheng et al., 2022), while quercetin supplementation significantly increased the relative abundance of *Akkermansia* and reduced the ratio of F/B in obese mice (Tan et al., 2021), improved LPS-induced inflammatory damage (Bian et al., 2018), maintained glucolipid metabolic homeostasis and promoting the production of SCFAs to enhance insulin sensitivity and improve the inflammatory response. Therefore, we hypothesize that quercetin may ameliorate reproductive endocrine disorders by reshaping the structure of the intestinal flora and increasing the content of SCFAs.

Curcumin is a major polyphenolic compound isolated from *Curcuma longa L* [Zingiberaceae; *Curcumae longae rhizoma*] with anti-inflammatory, antioxidant, anti-cancer and anti-microbial properties. It has been found that curcumin significantly reduces blood glucose, lipids and androgen levels, and promotes follicular development, maturation and luteinization in PCOS patients (Bachmeier et al., 2010; Jamilian et al., 2020). Moreover, curcumin reversed ovarian oxidative stress damage, improved ovarian reserve function, increased the number of primordial follicles, decreased the number of atretic follicles and increased AMH levels (Kevenaar et al., 2006; Wang X. N. et al., 2017; Yan et al., 2018). Curcumin has also been used in recent years in studies of EMs due to its anti-inflammatory, anti-angiogenic and anti-proliferative properties and its positive effects (Chowdhury et al., 2019). It was discovered that curcumin modulated the diversity and distribution of intestinal flora, partially reversed ovariectomy-induced estrogen deficiency in rats, improved intestinal barrier function and regulated gut-brain axis homeostasis, inhibited chronic inflammatory responses and improved insulin sensitivity, and prevented metabolic and reproduction-related diseases (Wang J. et al., 2017; Zhang et al., 2017; Di Meo et al., 2019). Resveratrol is a low molecular weight polyphenolic compound widely found in various botanical drugs, e.g. *Reynoutria japonica Houtt* (Polygonaceae; *Polygonum cuspidatum Sieb. et Zucc.*), fruits (including grapes and peanuts) and red wine that maintains homeostasis of glucolipid metabolism, improves low-level inflammation and oxidative stress, and is beneficial in the treatment of metabolic, reproductive and inflammatory proliferative diseases (Chaplin et al., 2018; Jiang et al., 2019). After resveratrol treatment, serum TNF-α and T levels were significantly reduced in PCOS rats, the degree of IR was alleviated, and follicular development was maintained (Furat Rencber et al., 2018). Resveratrol is also known as a natural anti-inflammatory agent, and it was found that resveratrol might act as an anti-inflammatory and anti-proliferative agent *via* multiple pathways such as arachidonic acid, NF-κB, Ah receptor or AP-1 to prevent EMs (Dull et al., 2019). Besides, resveratrol also upregulated PPAR-γ and SIRT1 expression, inhibited NF-κB-mediated inflammatory response, increased serum AMH levels and reduced ovarian inflammation, and restored ovarian reserve function (Said et al., 2016). Intestinal flora dysbiosis is an important pathological link in PCOS, EMs and POIs, and resveratrol has the effect of regulating the structure of intestinal flora, such as increasing the abundance of *Bacillus mimicus*, *Lactobacillus* and *Bifidobacterium*, and decreasing the abundance of *Firmicutes* and *Enterococcus faecium*. Therefore, we speculate that resveratrol may exert anti-inflammatory and anti-glycolipid disorder effects to prevent and treat female reproductive disorders by modulating the structure of intestinal flora, but further experimental studies are needed to verify this.

## 5 Discussion

Dysbiosis of intestinal flora is closely related to the development and progression of female reproductive disorders (Qi et al., 2021). The absence of beneficial bacteria and overgrowth of certain pathogens may be one of the important pathogenesis of female reproductive disorders. F/B ratio is considered as a marker of dysbiosis and is positively correlated with the levels of pro-inflammatory factors such as TNF-α, IL-1β, IL-18 and IL-6. A lower F/B ratio is thought to be beneficial in improving inflammation and maintaining pelvic microenvironment homeostasis (Chu et al., 2019). However, it is of concern that *Lactobacillus* is considered to be the most commonly used probiotic and a large number of studies have shown that Chinese herbal medicines can improve the symptoms of reproductive endocrine disorders by increasing such probiotics, but there are also individual studies that have shown a decrease in this genus during treatment with Chinese herbal medicines (Jiang X. L. et al., 2020; Deng et al., 2021). Therefore, attention should be paid in subsequent studies to correlate the possible functions of the genus with the pharmacodynamic indexes and to validate them in order to illustrate the significance of the changes in their abundance.

Botanical drugs, Chinese herbal formulas and active ingredients of Chinese herbal medicines (alkaloids, flavonoids, triterpenoids, polyphenols and polysaccharides) can effectively improve reproductive endocrine symptoms by regulating the diversity and composition of intestinal flora and its metabolites, SCFAs, indicating that the regulation of intestinal flora is one of the important channels for the treatment of reproductive endocrine diseases by Chinese herbal medicines. However, most current studies have only discussed the modulation of intestinal flora structure and composition, effects on intestinal immune inflammatory response and intestinal barrier function and on brain-gut axis by Chinese herbal medicines that improve reproductive function, while most studies on the mechanism of intestinal flora regulation by Chinese herbal medicines that alleviate reproductive dysfunction have only conducted association analysis studies, and further cause-effect studies are rare. Follow-up studies can be based on macro-genome sequencing technology to analyze the changes of intestinal flora structure under the intervention of Chinese herbal medicines in reproductive endocrine state, perform species function annotation, screen differential strains, and perform experimental validation by fecal transplantation or culture of specific flora based on specific flora regulated by Chinese herbal medicines that have been clearly identified in clinical and animal experiments to improve reproductive function. Then, correlation analysis was performed in combination with intestinal contents or fecal metabolomics, and validated by relevant pathway indicators, and the biological mechanisms of Chinese herbal medicines to improve reproductive endocrine function were explored in depth, with a view to elucidating the mechanism of action of Chinese herbal medicines in treating reproductive disorders.

There are a wide variety of components in Chinese herbal medicines and formulas, and the material basis of the effect of correcting reproductive disorders is not clear. Based on the study of crude extracts, active ingredients and compounds of Chinese herbal medicines, the reproductive protection mechanism of their active parts or groups of active ingredients can be further investigated. Numerous studies have confirmed that Chinese herbal medicines can affect the growth and composition of microbiota, and can also be metabolically inactivated or transformed by microbiota, thus directly or indirectly affecting the therapeutic effects of drugs. Chinese herbal medicines regulate the structure of intestinal flora, increasing the abundance of phylum *Bacteroidetes* and genus *Akkermansia*, *Bacteroides*, *Bifidobacterium*, *Prevotella*, and *Lactobacillus*, while decreasing the abundance of phylum *Firmicutes* and F/B ratio, and prevent or treat female reproductive disorders through SCFAs, BAs, and LPS signaling. However, the role of intestinal flora on the active ingredients of Chinese herbal medicines to improve reproductive disorders has yet to be explored in depth. Therefore, the interaction between the active ingredients of Chinese herbal medicines as well as compounded active ingredients and intestinal flora deserves attention, and *in vitro* co-incubation of the identified active ingredients with the corresponding metabolic flora can be considered to further investigate the material basis of the effect of Chinese herbal medicines on improving reproductive disorders, in order to elaborate the material basis research of Chinese herbal medicines for treating reproductive disorders diseases and provide a basis for the research of new Chinese herbal medicines for improving reproductive functions.

## 6 Conclusion

In this review, we explore the potential mechanisms by which Chinese herbal medicines are involved in improving female reproductive disorders and regulating the intestinal flora. Our review shows that Chinese herbal medicines increase the abundance of probiotic bacteria, such as *Akkermansia* to regulate intestinal integrity and barrier function, and SCFA-producing bacteria to improve host metabolism and inflammation. Meanwhile, Chinese herbal medicines can reduce the abundance of pathogenic bacteria and inhibit LPS-induced metabolic endotoxemia and inflammation. Moreover, some Chinese herbal formulas can increase the levels of GLP-1 and PYY by stimulating enteroendocrine cells, thereby regulating the gut-brain axis and affecting glucolipid metabolism, for example, Wang’s YiJing Decoction. The above still needs to be verified by further studies in the future. Furthermore, there are still some shortcomings in the treatment of female reproductive disorders in Chinese herbal medicines. For example, there are different treatment strategies due to the different classification criteria of TCM for female reproductive system diseases, a single and small number of clinical study samples, and a lack of high-quality multicenter large sample studies. It is not clear which herbal components play key pharmacological roles in regulating intestinal flora, and there are significant limitations in the promotion and use of Chinese herbal medicines. Therefore, more high-quality clinical studies on Chinese herbal medicines for female reproductive disorders are needed in the future, so as to establish a quantitative system of TCM efficacy and promote safer and more effective use of Chinese herbal medicines. More importantly, the correlation between intestinal flora and disease-specific metabolites should be studied in depth, while finding the active ingredients of Chinese herbal medicines and screening out the specific ingredients that act on the relevant intestinal flora or metabolites, so as to finally achieve precise treatment for patients with female reproductive disorders.
